# Supplementary material for: Shared Treg dysregulation underlies psoriasis vulgaris and atopic dermatitis through the PHF19-PRC2 epigenetic axis
Source: Front Immunol. 2026 Aug 20;17:1932788. doi: 10.3389/fimmu.2026.1932788 (PMC13538097; doi:10.3389/fimmu.2026.1932788)
Supplement: Supplementary file 3 [file DataSheet3.zip › Additional files/Additional file 1/51.NDUFA12.scatter.pdf]

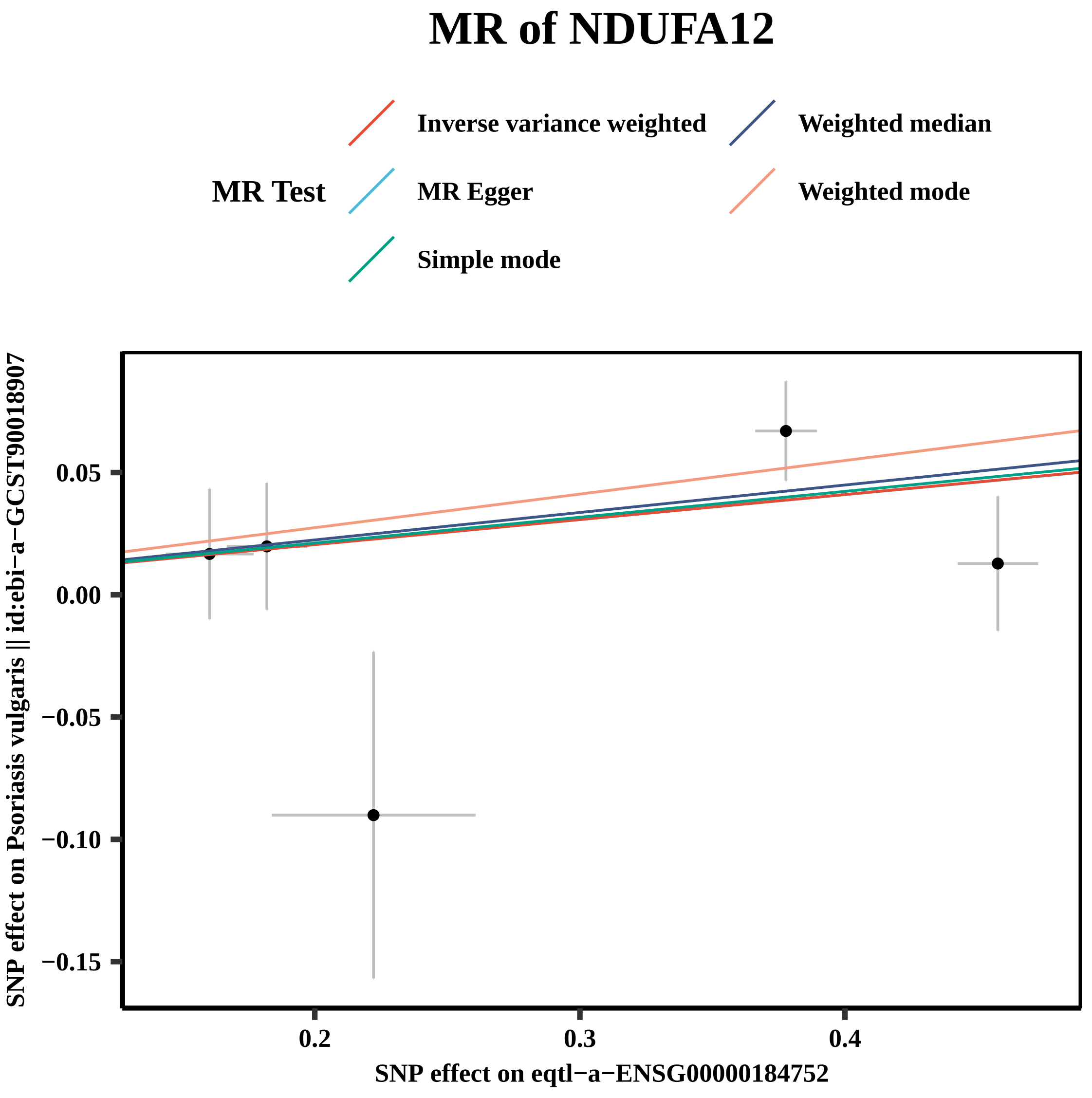
MR of NDUFA12
MR Test
Inverse variance weighted
MR Egger
Simple mode
Weighted median
Weighted mode
SNP effect on Psoriasis vulgaris || id:ebi−a−GCST90018907
0.05
0.00
−0.05
−0.10
−0.15
0.2
0.3
0.4
SNP effect on eqtl−a−ENSG00000184752
